# A century of National Forest Inventory in Norway – informing past, present, and future decisions

**DOI:** 10.1186/s40663-020-00261-0

**Published:** 2020-07-17

**Authors:** Johannes Breidenbach, Aksel Granhus, Gro Hylen, Rune Eriksen, Rasmus Astrup

**Affiliations:** National Forest Inventory, Norwegian Institute of Bioeconomy Research, Ås, Norway

## Abstract

**Past:**

In the early twentieth century, forestry was one of the most important sectors in Norway and an agitated discussion about the perceived decline of forest resources due to over-exploitation was ongoing. To base the discussion on facts, the young state of Norway established *Landsskogtakseringen* – the world’s first National Forest Inventory (NFI). Field work started in 1919 and was carried out by county. Trees were recorded on 10 m wide strips with 1–5 km interspaces. Site quality and land cover categories were recorded along each strip. Results for the first county were published in 1920, and by 1930 most forests below the coniferous tree line were inventoried. The 2nd to 5th inventories followed in the years 1937–1986. As of 1954, temporary sample plot clusters on a 3 km × 3 km grid were used as sampling units.

**Present:**

The current NFI grid was implemented in the 6th NFI from 1986 to 1993, when permanent plots on a 3 km × 3 km grid were established below the coniferous tree line. As of the 7th inventory in 1994, the NFI is continuous, and 1/5 of the plots are measured annually. All trees with a diameter ≥ 5 cm are recorded on circular, 250 m^2^ plots. The NFI grid was expanded in 2005 to cover alpine regions with 3 km × 9 km and 9 km × 9 km grids. In 2012, the NFI grid within forest reserves was doubled along the cardinal directions. Clustered temporary plots are used periodically to facilitate county-level estimates. As of today, more than 120 variables are recorded in the NFI including bilberry cover, drainage status, deadwood, and forest health. Land-use changes are monitored and trees outside forests are recorded.

**Future:**

Considerable research efforts towards the integration of remote sensing technologies enable the publication of the Norwegian Forest Resource Map since 2015, which is also used for small area estimation at the municipality level. On the analysis side, capacity and software for long term growth and yield prognosis are being developed. Furthermore, we foresee the inclusion of further variables for monitoring ecosystem services, and an increasing demand for mapped information. The relatively simple NFI design has proven to be a robust choice for satisfying steadily increasing information needs and concurrently providing consistent time series.

## Introduction

Statistics on forest resources and their future development are pivotal to making informed decisions in times of a changing environment. In most of the developed world, we today take for granted to have this form of information provided by National Forest Inventories (NFIs) (Tomppo et al. 2010; Vidal et al. 2016). Indeed, governments were only willing to bear the costs and ongoing commitment of an NFI system because the lack of reliable statistics on forest resources became untenable. The first NFIs were implemented in the Nordic countries due to a fear of overexploitation of the forest resource. Norway started with institutional settings and planning in 1917, and field-work in 1919 (Landsskogtakseringen 1920), while Finland and Sweden followed in the early 1920s (Ilvessalo 1927; Thorell and Ostlin 1931; Fridman et al. 2014; Kangas et al. 2018). The USA implemented a forest monitoring program in the late 1920s. The fear of forest decline due to acid rain in the 1980s was among the reasons for the implementation of the NFI in West Germany - despite the availability of detailed local forest management inventories (Kleinn et al. 2020 in press). Incentives in the form of development aid and information requirements resulting from mitigation of climate change (REDD+) resulted in the establishment of NFIs in a number of developing countries (e.g. Tomppo et al. 2014; Tewari and Kleinn 2015).

The main motivation of the first NFIs was to provide statistics on the main dendrometrical variables such as timber volume and volume increment, to guide political decisions that guaranteed a sustainable management of the timber resource. Today, NFIs have gradually developed to include many other ecosystem services and often are landscape inventories covering the land-based resources of a country. The Norwegian NFI, like many others, is for example used to estimate the areas and changes among the land use categories like forest, grassland, croplands, wetlands, and settlements that are required for reporting under the Climate Convention (UN 1992). However, the first Norwegian NFI already mapped other land resources than forest such as grassland, cropland, and mires. The clear aim of this setup was to support decisions on afforestation activities for example by the drainage of mires. In subsequent inventories, the description of the production potential of existing forest was made to support decisions on how low-productive forests could be replaced with forests consisting of more productive tree species. To provide useful information, estimates had to be reliable for rather small administrative units. All historic Norwegian NFIs until the 1990s had sample designs that allowed reliable estimates at least on the level of Norway’s 20 counties. Later, additional data have been collected on temporary plots to periodically provide county-level information. The second Norwegian NFI even provided reliable estimates for municipalities which, besides the war years, was the reason for its long duration from 1937 to 1956. Municipal-level inventories based on sample plots additional to the national grid were common until the mid-1980s (Tomter 2019, p. 186).

While the provision of small-scale estimates was less in focus for the Norwegian NFIs towards the end of the twentieth century, the lack of this information was one of the main reasons for developing the forest resource map SR16 which is based on a combination of NFI field plots and 3D remotely sensed data (Astrup et al. 2019). SR16 provides maps of the main forest characteristics such as biomass, timber volume, and tree species and is used for small area estimates on municipal level (Breidenbach and Astrup 2012). When overviewing a long NFI history, the repeated appearance of other questions also becomes visible, albeit in a new context to address present-day challenges. For example, NFI data were recently used to estimate the climate mitigation potential of replacing low-productive unmanaged deciduous forests with more productive coniferous forest (Bright et al. 2020). While this topic was of great interest after World War II because of the aim to increase timber production, it is today again under discussion in Norway as one option to increase the CO_2_ sequestration of forests. Another example is the implementation of sampling designs that allow annual estimates, which was already implemented in the fourth NFI from 1964 to 1976. This design marks a stronger emphasis on national estimates than on local to regional estimates. Since the mid-1990s, this concept is used again as the NFI became a continuous annual program, which was needed for the annual reporting to Statistics Norway.

The technological and societal changes over the last 100 years in Norway naturally led to the development of the NFI. The increasing availability of topographical maps, albeit coarse by today’s standards, determined the order in which counties were inventoried in the first NFI. Strip sampling was the most cost-effective way to gain information because of lack of infrastructure and modern forms of (air-) transportation (Kleinn and Tomter 1993). Sample plots were used after World War II, when the forest road network expanded rapidly, and motorized transportation became the standard. The development of inventory techniques was utilized to make the field data collection more efficient over time. Besides fixed sample plots, concentric sample plots and angle count sampling (ACS) (Bitterlich 1948) were also utilized through the years. Today, elements of fixed sample plots, concentric sample plots, ACS, cluster sampling, and line intersect sampling are utilized to assess the various variables. The technological development of motorized transportation, satellite-based navigation, emergency localization, mobile communication, hand-held computers, and ultra-sonic measurement devices resulted in the gradual reduction of field teams from 6 to 9 persons in the first NFI to just one since around 2000.

The increased information requirements of modern societies are also reflected in the Norwegian NFI. The number of recorded items has increased over the years to more than 120 tree-, stand-, and landscape-level variables. The creators of the first NFI certainly would be excited to hear that the NFI today also fulfills governmental reporting duties under various international treaties such as the global Forest Resource Assessment (MacDicken 2015) or the Climate Convention. The reporting requirements of the latter also resulted in a design change as from 2005. The forest in low-productive alpine regions, typically dominated by mountain birch, was also then included in the sampling frame and the NFI today covers the whole Norwegian land area. The currently implemented design proved to be robust for the required adjustments so far.

**Fig. 1:**
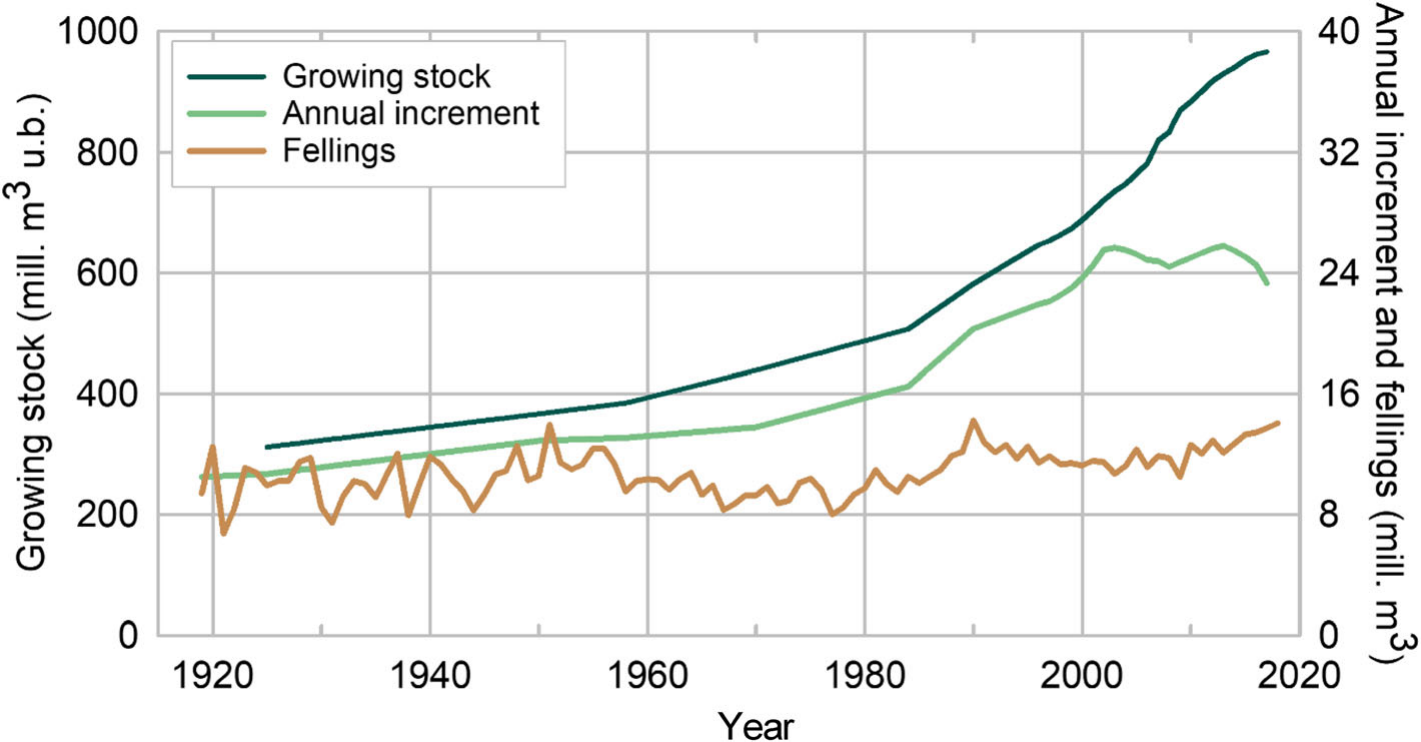
Growing stock volume under bark, annual increment and fellings for the period 1919–2017. Standard errors for volume and increment are in the order of 1% and 2%, respectively. Fellings includes the volume of all trees, living or dead, that are felled, including the volume of trees or parts of trees that are not removed from the forest. The volume of removals of round wood and firewood for commercial use and household consumption are based on a combination of census and sampling surveys by Statistics Norway (SSB). Removals are converted into fellings by adding estimated harvesting losses

The first Norwegian NFI showed that increment and harvest were approximately in balance (Fig. 1). This calmed the discussions regarding the fear of over-exploitation of the forest resource (Barth 1916). The NFI documents an increase of the growing stock timber volume by a factor of three over the last 100 years (Fig. 1). Many policy decisions, often informed by NFI statistics, had an influence on this result. Among them were the change of the silvicultural system to even-aged stand management with active reforestation after harvests, afforestation, breeding of improved seed material, and institutionalized research and education. One of the main drivers for this development was, however, the substitution of wood as the primary energy source by fossil fuels. In recent decades (Fig. 1), the feed-back effects of the large fossil fuel combustion are becoming visible in an increased increment attributed among other things to increased N-deposition (Solberg et al. 2004) and longer growing seasons due to climate change (Nordli et al. 2008).

Despite its long history, only a few international accounts of the Norwegian NFI system have been published (Landsskogtakseringen 1968; Kleinn and Tomter 1993; Tomter et al. 2010; Tomter 2016). Our aim is therefore to describe developments in the past, present, and foreseeable future design and use of the Norwegian NFI. By sharing experiences gained throughout the years, we hope to contribute to the ongoing development of the Norwegian NFI and other NFIs around the globe. In the remainder of the text, we will use the acronym NFI to refer to the Norwegian National Forest Inventory, unless otherwise specified.

## The historical development of the NFI

### Historical background

The description of the historical background is based on books in Norwegian that were published to celebrate anniversaries of the NFI and the organizations hosting the NFI at the time (Landsskogtakseringen 1970; Strand 1994; Øyen 2006; Tomter 2019). The reports of the early NFIs were valuable resources regarding the applied methodology (Landsskogtakseringen 1920, 1933).

Reports on local overexploitation of forests date back to the sixteenth century when also the first laws regulating timber export and the establishment of saw mills were enacted. Besides the still typical use of timber for construction, huge amounts of firewood were required in households and in the dairy production on alpine summer farms in many regions. Domestic animals foraging near the summer farms also led to local forest degradation and even a lowering of the tree line compared to the present-day level (Bryn and Daugstad 2001; Bryn and Potthoff 2018). Charcoal was an important energy source in Norway’s early industry and was used in e.g. mining and processing of iron, copper, and silver.

Aiming at protecting the regeneration of forests, high-grading was enforced as the forest management regime, where the harvest of trees below a certain diameter was strictly forbidden. Due to the high timber demand, the fastest growing trees had a high likelihood to be harvested first, which over time may have contributed to a slower growth of the forests. High-grading was still the prevailing management regime when Barth (1916) published an influential article where he predicted the doom of the Norwegian forest. It included a rough estimate according to which the harvest levels were 29% higher than the increment.

Initial ideas of obtaining national forest statistics date back to 1737, when Norway’s first forest administration was founded. The commitment of the king that ruled Denmark-Norway from the Danish capital Copenhagen was, however, not strong enough to implement such a revolutionary task. During the nineteenth century, a growing population together with the release of sawmilling privileges in the 1860s and a growing pulpwood industry increased the timber demand and led to local timber shortage. The increasing utilization of the forest resources generally took place without knowledge on whether the harvest was sustainable, although some attempts were made to estimate volume increment and removals (Skogkommisjonen 1874; Helland 1894). The problem for those estimates was that they were not authoritative because uncertainties remained unknown and the need of better forest statistics was repeatedly formulated by the forest administration.

While it is unknown to us since when local forest management inventories have been conducted in Norway, a case from the municipality Åmot is documented. The inventory was started in 1907 by using 0.1 ha sample plots that were subjectively located in representative stands. By 1909, the design was changed to strip sampling to finalize the inventory of the northern part of the municipality. At a meeting of the Swedish Forestry Association in 1909 on methods of obtaining information on volume and increment in all of Sweden’s forests, participants of the forest inventory in Åmot reported from their experiences. Swedish representatives also visited Åmot around that time. Using strip sampling, they conducted an inventory of Värmland county in 1911–1912 as a pilot study for the whole of Sweden (Anon 1914).

The Norwegian State Forest Inventory (SFI) had the duty to conduct forest management inventories of the forests owned by the Norwegian state and started using strip sampling around 1914. When the director of Statistics Norway, Nicolay Rygg, met with experts of the SFI in 1915, he had since several years plans to get more reliable information on the Norwegian forest resources. At the meeting, Rygg got to know about the inventory of Värmland in Sweden and the experiences of the SFI gained in Norway. Together with experts from the SFI, he developed a preliminary plan for an NFI in Norway which he brought forward to the prime minister Gunnar Knudsen. The positive response of the prime minister resulted in funds to elaborate a plan and budget for an NFI as a governmental institution, which was accepted by a parliamentary decision on 13 June 1917.

### The first NFI

**Fig. 2:**
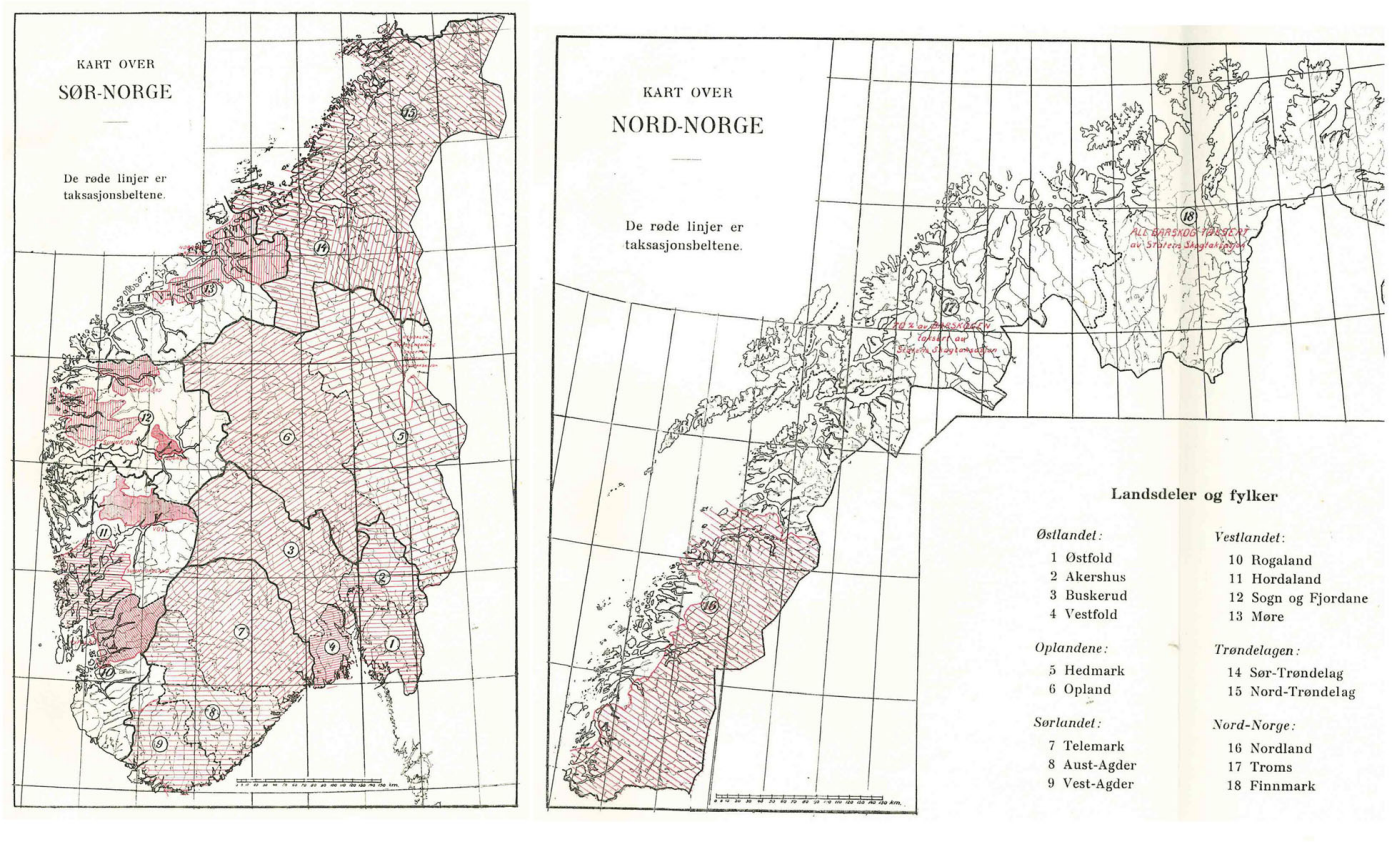
Maps of southern and northern Norway showing the coverage and strip density of the first NFI (source: Landsskogtakseringen 1933). Numbers: Inventory areas (counties). Red lines: Inventory strips

The Norwegian NFI – *Landsskogtakseringen* – was formally established on 1 June 1919, when a leader and assistant were employed that were responsible for conducting the first NFI. They were supported by a commission consisting of three inventory experts. Right after his employment in June 1919, the leader of the NFI visited the Swedish forest research institute to study the Värmland inventory, which had a big influence on the NFI design. Field work started in August 1919 in Østfold county and carried on by county until field work was finalized in 1930, while county-level reports were published in the meantime (e.g. Landsskogtakseringen 1920). All counties were fully covered except for western and northern Norway. As can be seen from Fig. 2, only large contiguous areas with forest were included in western Norway due to the low forest cover in large parts of that area. The two northernmost counties Finnmark and Troms and an area around Rendalen in eastern Norway were not covered by the NFI because most of these regions were state-owned and had been inventoried by the SFI (Landsskogtakseringen 1933, p. 28). As for all following NFIs until 2005, the surveyed population was the area below the coniferous forest limit.

#### Field measurements

Field work was carried out along 10 m wide strips that were oriented perpendicular to the main valleys in the respective county or watershed (Fig. 2). The distance between the strips was between 1 and 5 km. Each strip was split into 2 km long segments that were treated as a 2 ha sampling unit. Within forest, the number of trees ≥15 cm dbh within 5 cm classes and the tree species groups spruce, pine, and deciduous were recorded. On the right half of the strip, trees ≥5 cm were also recorded. Trees between 0 and 5 cm were recorded on the right half of the strip for the easternmost 100 m of every kilometer.

Sample trees were selected according to proportions that were determined beforehand by the headquarters. The proportions varied per county and diameter class. For the smallest trees, every 50th to 300th tree was selected while every 3rd to 10th tree with a dbh ≥45 cm was selected. Tree species, distance to the center line, dbh in mm, diameter at 5 m height, total height, form height (Jonson 1915), damage, the latest decade’s height growth, the latest decade’s dbh growth, and age were recorded for sample trees. The increment cores taken for the latter two variables were collected for laboratory analysis.

An assessment of the site index (SI) was made by dividing the strip segments within forest into classes of high, medium and low productivity. In addition, the class protection forest, mostly towards the alpine border, described sensitive forests that would become non-forest if too strongly harvested. Areas below the tree line were classified as productive forests, agriculture, extensive grasslands, forest pastures, mires, water, settlements, and barren land due to unfavorable growth conditions. Mires were categorized according to their suitability for drainage and all land was categorized according to the possibility for converting it into cropland. The classification was largely based on expert judgement.

The field teams consisted usually of at least six people: a team leader, a writing clerk, a navigator, two men doing measurements and following the strips, and one man doing the sample tree measurements. While the team leader had to have a higher forestry education, the other team members usually had college or high school education with a forestry major. At the end of each week, which consisted of at least 48 working hours, the completed forms for each segment and a report with a description of the progress and a place where the field crew could be found, were sent by mail to the headquarters.

#### Office work

Sample tree volumes without bark were predicted by multiplying the tree’s basal area with its height and form factor (Landsskogtakseringen 1933, p. 30). The form factor method by Tor Jonson was obviously very common at the time and therefore not further documented but most likely refers to Jonson (1910). Volume and increment with bark were predicted using Swedish volume tables by Tor Jonson (most likely: Jonson 1915). Mean-tree volume and increment were estimated for each dbh class in the whole county. The number of trees was estimated by multiplying the recorded tree numbers with the sampling fraction of the strips. The estimated number of trees was multiplied with the mean tree volume and volume increment from the sample trees to estimate total volume and increment.

Parallel to the NFI, a Census Survey of Forest owners (CSF, *Skogbrukstellingen*) was conducted by Statistics Norway 1920–1927, which provided information on harvest removals and forest ownership structure. The results of the first NFI and CSF showed that, over all, there was a balance between increment and harvests. Data from the NFI, SFI, and CSF were combined for a complete assessment of Norway’s forests. The difference compared to earlier attempts at obtaining forest statistics was that uncertainty could be estimated. Following the example of the Swedish inventory of Värmland, simple random sampling within each of 10 groups of 2 km segments was assumed for variance estimation in the beginning. The variance estimates of the 10 groups were then added to obtain a variance for the whole area (Landsskogtakseringen 1920, p.89). Difference estimators developed by Langsæter (1926) and his Nordic colleagues (Lindeberg 1924; Näslund 1930), were utilized for the national estimates (Landsskogtakseringen 1933, p. 112). The standard errors for national forest area, growing stock volume, and increment were 0.48%, 0.74%, and 0.77%, respectively.

### The following NFIs until the 1990s

The second NFI in 1937–1956 aimed at providing reliable estimates for municipalities or groups of municipalities. Strip sampling was used until 1956 with varying strip interspaces as small as 600 m. As of 1956, temporary circular sample plots with an area of 78 m^2^ were used that were laid out on grids of 2–3 km × 200 m. In one county, a 3 km × 3 km grid was used with clusters of 20 plots at each point. A more objective site index system with five categories was introduced. Finnmark county, the northernmost part of Norway, was not included in the NFI until 2005. The second NFI introduced a system to describe the developmental stage and the state of forest stands based on Alf Langsæter’s system of five maturity classes, which modified a Swedish system (Landsskogtakseringen 1938; Esmark 1965). This system was used with some adjustments until 1957, when the system as we know it today was implemented (Section “The maturity class system”) (Landsskogtakseringen 1959).

The third NFI in 1957–1964 aimed at relatively quickly providing updated information for larger regions on the background of new fears of overexploitation of Norwegian forests after World War II. For most parts of the country, square clusters of 20 plots on a 3 km × 3 km grid were used. The distance among plots in a cluster was 200 m and the circular plot area was 100 m^2^. Several new variables were recorded, among them stand type with respect to tree species mix, vegetation type, terrain properties of interest for timber harvest, and soil type and depth.

The fourth NFI in 1964–1976 used the same design as before, with the difference that the clusters were not measured per county. Instead, each year 1/12th of the clusters distributed over the whole country were measured to provide national estimates quickly. Western Norway (and Finnmark) were not covered by the NFI.

The fifth NFI in 1980–1986 was mostly based on clusters of 12–16 angle count plots. The counties in western Norway were included with clusters mostly on a 3 km × 3 km grid. The smallest estimation unit for the area that was covered by the fourth NFI were regions consisting of 2–3 counties where the grid size varied between 4.8 km × 3.2 km and 7.2 km × 6.4 km. At that time, the NFI conducted various municipal-level inventories by increasing the plot density locally.

The sixth NFI in 1986–1993 established permanent sample plots on a 3 km × 3 km grid below the coniferous forest limit that are still used today. Clustered with each permanent plot were 3–11 temporary sample plots at distances of 250–500 m for county-level estimates (Section “County inventories”). On the concentric sample plots, trees with a dbh ≥5 cm were measured on a 100 m^2^ plot, while trees with a dbh ≥20 cm were measured on a 200 m^2^ plot. As of 1988, all data were recorded in field computers instead of on paper.

Since 1984, a tree crown condition survey was performed on all sample trees in the NFI due to alarming reports of “novel forest damage” in central Europe (ICP Forest 2016). As of 1988, a variety of crown condition variables were assessed annually on a subset of NFI plots located on a 9 km × 9 km grid for Norway spruce and Scots pine, and on a 18 km × 18 km grid for birch (Aamlid et al. 2000). A soil survey was carried out for these plots 1988–1992 (Esser and Nyborg 1992; Esser 1994) which included a description of a representative soil profile and a soil sample for chemical analysis. A sub-sample of plots was resampled after 5–6 years (Esser 1996). As of 2013, annual assessments were stopped, and the crown condition survey was fully integrated in the 5-year NFI cycle.

## Institutional settings throughout time

For more than 50 years, the NFI was an independent institution reporting directly to the Ministry of Agriculture, with headquarters in Oslo. In December 1972, the NFI became a branch of the Norwegian Forest Research Institute (NISK) located in Ås, and remained so until January 1988, when the tasks and staff were transferred to the Norwegian Institute of Land Inventory (NIJOS), which was established at the same time. In 2006, the forest research and land inventory institutes were merged to form the Norwegian Forest and Landscape Institute, and the NFI was organized as a section within the new institute. Since July 2015, the NFI has been organized as a Department within the Norwegian Institute of Bioeconomy Research (NIBIO), which was formed after a merger of the Forest and Landscape Institute with two other institutes that year. The Norwegian NFI is one of very few in Europe that is not anchored in a national law (Polley 2020). Since 2019, the field staff of the NFI are permanently employed in 24 full- or part-time positions. Previously, most of the field staff were temporarily hired for the field season.

## The present NFI system

### The sample design

Since the 7th inventory in 1994, the NFI is continuous, and 1/5 of the plots are measured annually in an inter-penetrating panel design. Due to the information requirements of the greenhouse gas reporting, the NFI grid was expanded in 2005 to cover the alpine region (above the coniferous tree line). For the same reason, the northernmost county Finnmark was for the first time included in 2005. Since the last sample plots in Finnmark were established in 2011, the sampled population is all of Norway’s mainland area including lakes. The annually updated field manual has more than 200 pages (e.g. Viken 2018) and is the most detailed reference of the methods used in the NFI. Here, we only describe a selection of the used methods and recorded variables.

**Fig. 3:**
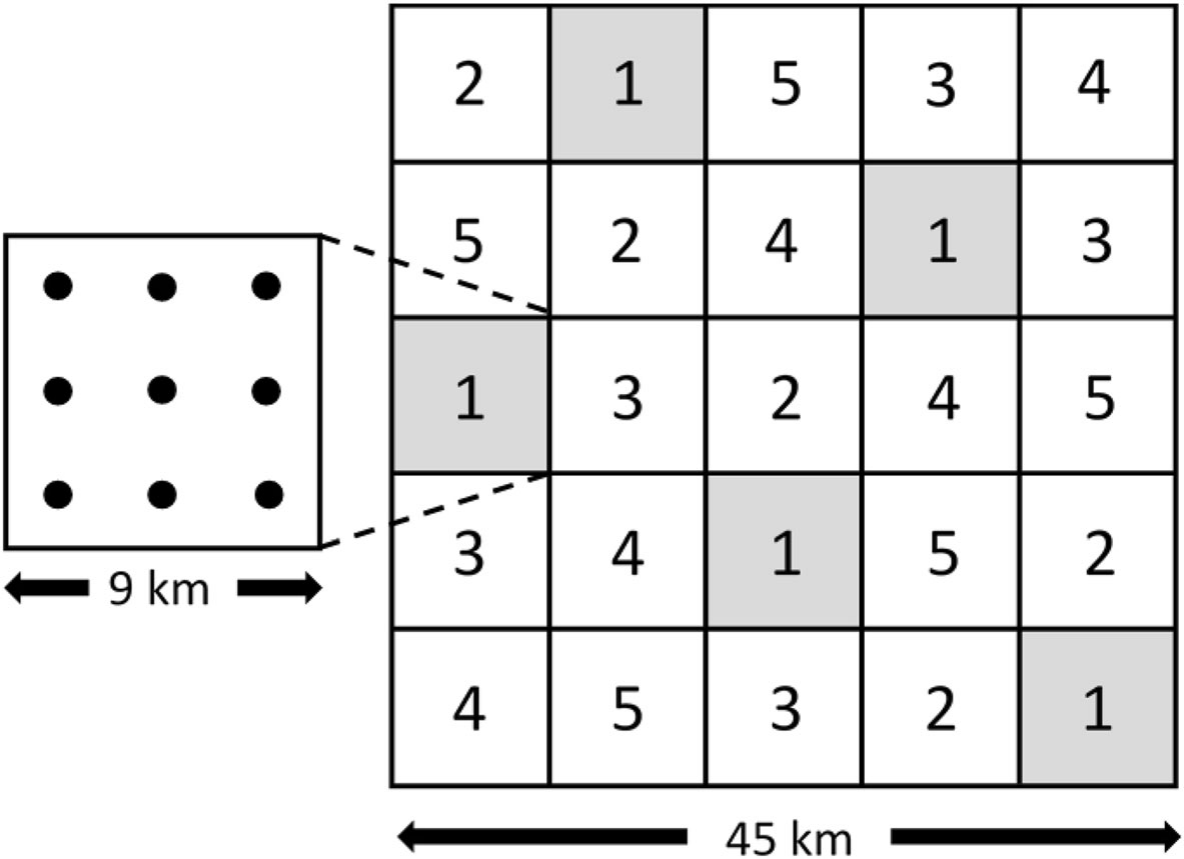
The Latin square design of the NFI. The numbers in the blocks indicate the panel of plots measured in 1 year. The blocks belonging to panel #1 are grey to visualize that blocks in one panel do not share borders. Nine sample plot locations on a 3 km × 3 km grid are located within each block

The permanent sample grid over all land use classes that is used for national estimates consists of 22,008 plots. The annual panel (i.e. 1/5 of the plots) is checked on the background of aerial images in the office. If a plot is assumed to contain trees, or if it was previously registered in the field, field staff are sent to the plot to make measurements. This includes plots that do not meet the forest definition. To reduce the influence of topography, Norway is tessellated into Latin squares. Each Latin square consists of 5 × 5 = 25 blocks, each with an area of 81 km^2^ (Fig. 3) (Viken 2018, p. 9). Each block contains 9 plot locations on the 3 km × 3 km grid. The panel of 1 year consists of the plot locations within one block of each row of the Latin square. The arrangement of plots in blocks of a Latin square has the dual advantage that it reduces travelling costs by concentrating the plots within a panel in blocks, while assuring that no adjacent blocks are within the panel of plots of 1 year.

**Fig. 4:**
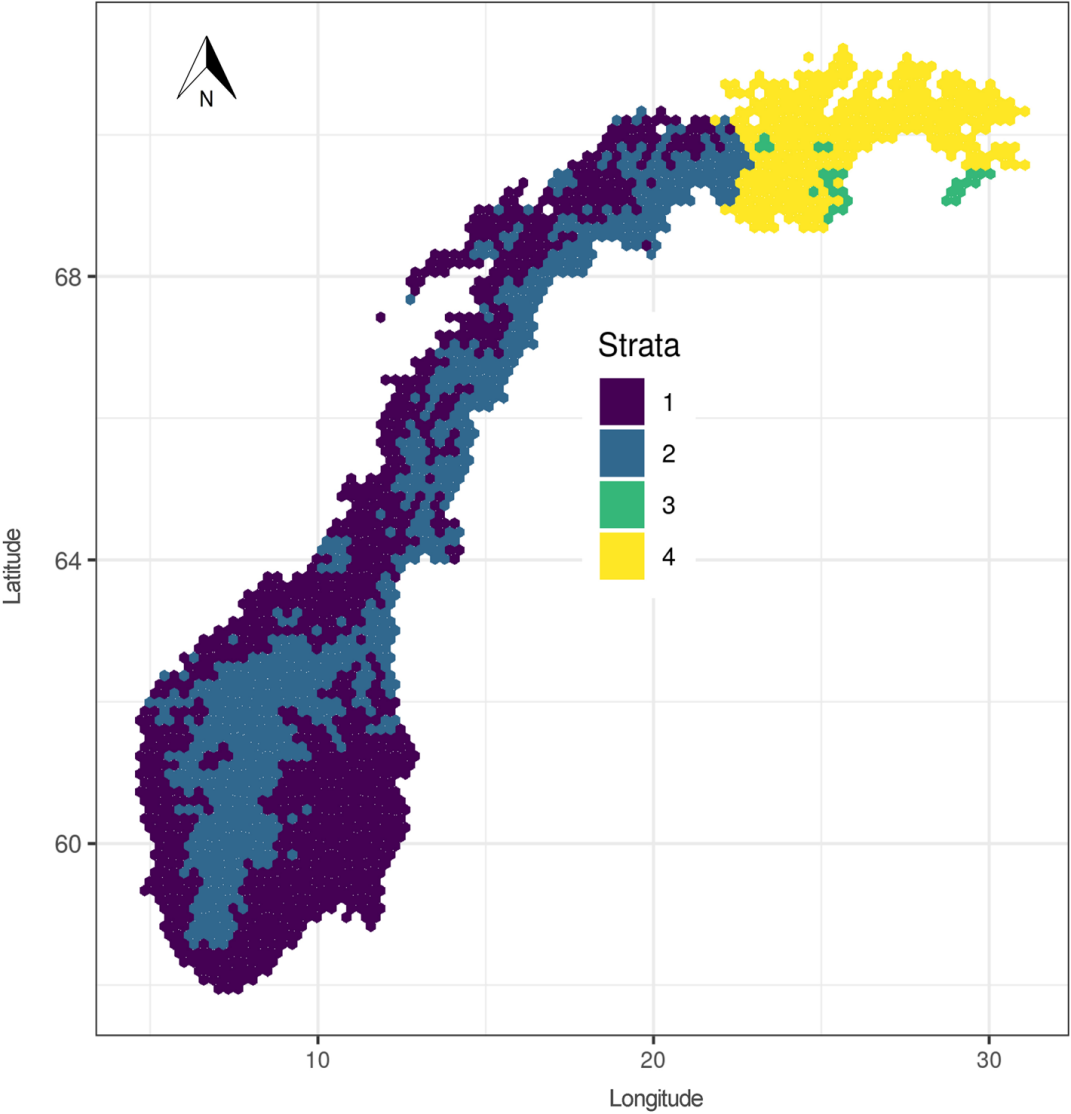
NFI stratum map (aggregated to large hexagons for display purposes)

Stratification by adjusting the grid size is often used to sample more observations in productive areas than in low-productive areas. In the NFI, a denser sampling grid is used in regions where conifers can be of economic interest. The northernmost county of Finnmark was not included in the NFI before 2005. Therefore, four design strata are utilized in the NFI; two in Finnmark (approximately east of longitude 22° east), and two outside Finnmark that cover most of Norway’s area (Fig. 4). For simplicity, we will refer to the latter as being located south of Finnmark. The sampling grid in the area south of Finnmark has a size of 3 km × 3 km in the lowland region (stratum 1) and 3 km × 9 km in the low-productive alpine region (stratum 2). In the latter, coniferous trees and active forest management seldom occur (Table 1).

**Table 1:** Forest area by dominant tree species group in the four NFI strata and all of Norway based on the NFI cycle from 2014 to 2018

Stratum	Spruce (%)	Pine (%)	Deciduous (%)	Unstocked (%)	Area (km^2^)	Forest (%)	# plots in forest	Forest area (km^2^)
1	34.0	33.7	31.8	0.6	149,885	66.4	11,046	99,551
2	3.6	8.1	87.8	0.5	125,281	9.5	442	11,906
3	0.0	64.2	35.8	0.0	1350	80.0	120	1080
4	0.0	4.2	94.9	0.8	47,266	20.5	118	9683
Norway	28.0	29.1	42.3	0.6	323,782	37.7	11,726	122,220

Stratum 1 covers approximately 46% of Norway’s land area and more than 80% of the country’s forest. In Finnmark, a 3 km × 3 km grid is used within regions where coniferous trees, which in Finnmark is mainly Scots pine, can form stands of economic interest (stratum 3) and a 9 km × 9 km grid is used outside these regions (stratum 4). As opposed to the rest of the country, the coniferous region is not mainly determined by elevation but by local topography. While spruce, pine, and deciduous forests cover approximately equal parts in stratum 1, almost 95% of the forests in stratum 4 are dominated (in terms of volume proportion) by deciduous trees. The forest proportion in the different strata ranges from 9.5% to 80.0%, of which up to 0.8% are unstocked areas (i.e. regeneration areas). Although the two coniferous species Norway spruce and Scots pine stand for the majority of biomass in Norway (42% and 30% respectively), areas dominated by deciduous trees (especially birches *Betula pubescens* Ehrh. and *B. pendula* Roth.) make up the largest fraction (42%) of the forest area (Table 1). Because the sampling fraction differs among the strata, the plots represent different portions of Norway’s land area (sampling weights). The sampling weights for plots in strata 1–4 are approximately 9, 27, 9 and 82 km^2^.

While the Finnmark strata 3 and 4 are based on a map of productive and non-productive forest, the decision on whether a plot belongs to stratum 1 or 2 was taken differently. For the municipalities along the west coast and in northern Norway (north of the Arctic Circle), areas above a certain elevation threshold were defined as alpine. These thresholds range from 100 to 800 m a.s.l. and were defined in cooperation with the local forest administration. Many municipalities in southern and south-eastern Norway do not contain high mountains. For the remaining area (approximately 40% of the country), the stratum allocation was made by the field staff that visited each sample plot on a 3 km × 3 km grid. If a plot showed characteristics of the alpine zone, this was recorded, and the plot was only included in the sample if it aligned with the 3 km × 9 km grid. For this area, a stratum map based on universal Kriging using logit-transformed elevation as the covariable was created to utilize remotely-sensed data in estimates. A spherical variogram model used to this end had a zero nugget, a range of 5600 m and a sill of 0.73. The model was used to interpolate among the full 3 km × 3 km grid with stratum information at each plot to create a 16 m × 16 m resolution map aligning with the forest resource map SR16 (Astrup et al. 2019).

### Field measurements

**Fig. 5:**
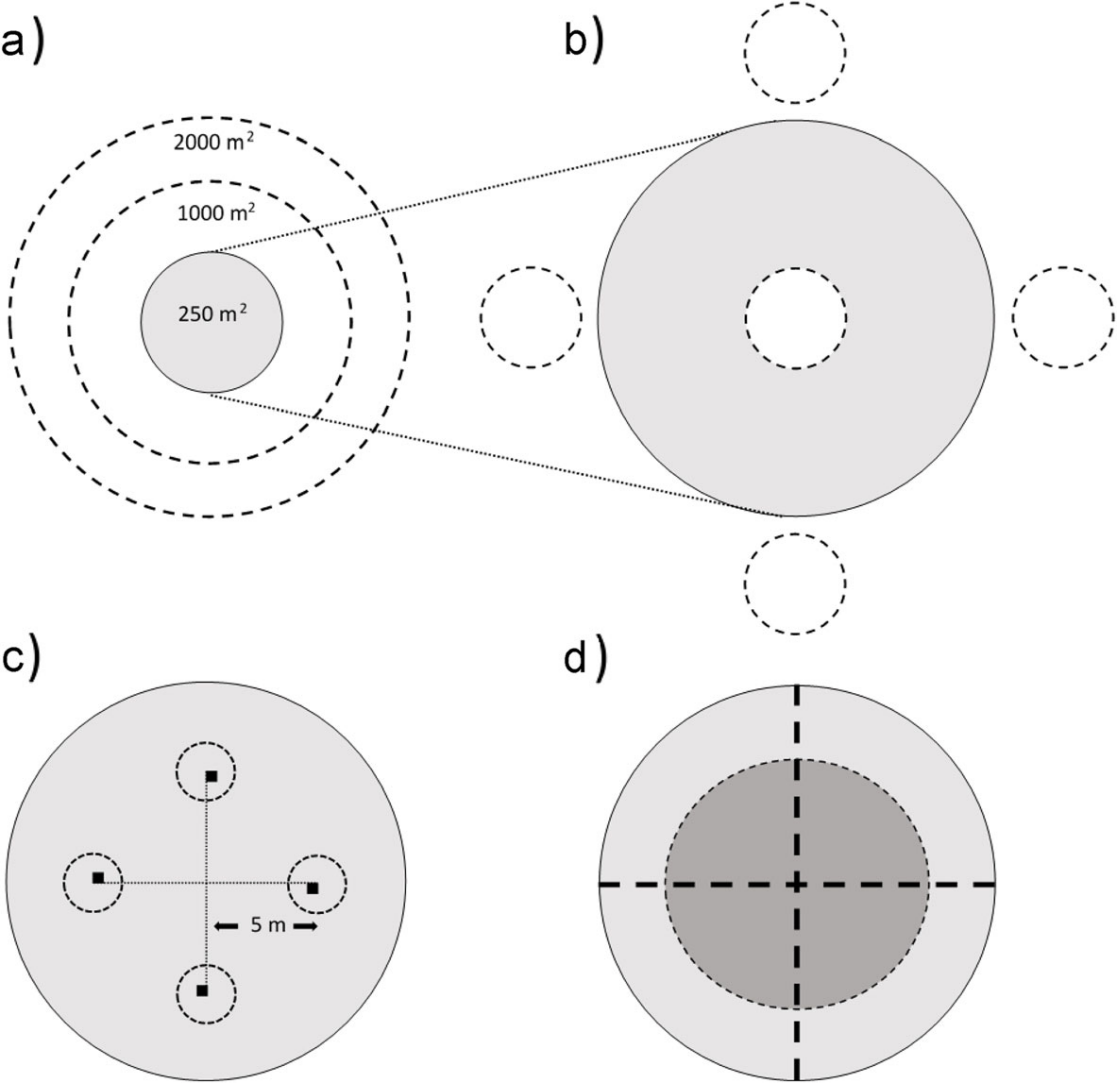
Design of the permanent plots. **a** Dendrometric variables for individual trees are measured on a plot sized 250 m^2^, here indicated in grey, while landscape- and stand-level variables, and biodiversity habitats are recorded on larger plots of 0.1 and 0.2 ha, respectively. **b** Tree densities and tree heights in young stands are measured on five 16 m^2^ subplots (dotted circles). **c** Four subplots are used to assess cover of bilberry and lingonberry (black squares), and for recording the number of trees with dbh < 5 cm and browsing intensity (dotted circles). **d** Patches of droppings from moose and red deer are counted on a 100 m^2^ plot (dark grey), while downed deadwood is measured along perpendicular transects of length 18 m (thick dotted lines)

Different sampling units are used to record the various variables in the field (Fig. 5):

- Variables such as growing stock volume, biomass, or increment are based on tree-level measurements on circular 250 m^2^ plots (8.92 m radius) (Fig. 5a). The plot center is marked with a metal stick buried in the ground and the center coordinate is measured using a handheld GPS. In addition, for 74% of the sample plots in forest so far, the coordinates have been measured using survey-grade GNSS equipment in an effort to measure all center coordinates within the next years. If the sample plot center is located close to a stand border, the plot is split into a maximum of two parts, if the stands are markedly different with respect to growing stock volume, maturity class (Section “The maturity class system”) or site index. Plots are also split if one part covers a different land-use category. In any case, the smaller plot part must have an area of at least 37.5 m^2^ (15% of the total plot area) to split a plot. Assessments of variables are carried out for each plot part. In addition to the tree-level measurements, some other variables like the dominant vegetation type and soil depth are assessed on the 250 m^2^ plot.
- Stand- and landscape-level variables such as forest type (dominant tree species), stand age, site index, maturity class (Section “The maturity class system”), soil type (mineral or organic), and land-use category are recorded on 0.1 ha circular plots concentric with the 250 m^2^ plot (Fig. 5a). If the 250 m^2^ plot is split, variables are assessed on two circle-segments, maintaining the assessment area for each plot part at 0.1 ha by adjusting the radius of the circle segments correspondingly.
- Biodiversity key habitats (Section “Assessment of key habitats on 0.2 ha plots”) are recorded on 0.2 ha circular plots concentric with the 250 m^2^ plot (Fig. 5a).
- In young forests (maturity classes 1 and 2, see Section “The maturity class system”), regeneration is assessed on five 16 m^2^ circular sample plots (2.26 m radius) located concentric with the plot center and 12 m in the cardinal directions from the plot center (Fig. 5b). Among other things, species and the number of trees > 30 cm are recorded.
- Small trees (dbh ≤5 cm) are assessed on circular 5.3 m^2^ plots (1.3 m radius) located 5 m from the plot center in the cardinal directions. Tree species, the number of trees in two diameter classes, and browsing damages are recorded (Fig. 5c).
- The ericaceous shrubs bilberry (*Vaccinium myrtillus* L.) and lingonberry (*Vaccinium vitis-idaea* L.) are important food sources for wild animals and resources for berry picking. Their coverage is recorded on 0.25 m^2^ squares located 5 m from the plot center in the cardinal directions (Fig. 5c).
- Downed deadwood is assessed along two 18 m long east-west and north-south oriented transects through the plot center (Fig. 5d).
- The number of moose (*Alces alces* L.) and red deer (*Cervus elaphus* L.) dropping patches are recorded on a 100 m^2^ plot concentric with the plot center (Fig. 5d).

As of today, more than 120 variables are recorded in the NFI including bilberry cover, drainage status, deadwood, occurrence of root rot on stumps, and forest health (Viken 2018). In the following sections we will describe some of the sampling units and variables in more detail.

#### Dendrometric variables on 250 m^2^ plots

##### Tree-level measurements

Diameter at breast height (dbh), tree species, vitality status (dead or alive), and polar coordinates are recorded for all trees with a dbh ≥5 cm. Predictions of volume, biomass, and increment for these trees are utilized in the national estimates of these variables. A caliper with the scale pointing to the plot center is used to measure dbh. At 1.3 m height, a (discrete) mark of red spray paint is applied such that subsequent measurements can be done at the same height. Experience from plots close to hiking trails shows that the marks are only visible to those that know they exist. The measured dbh is manually recorded in the field computer which selects a subsample of height-measurement trees, denoted h-trees.

Tree heights are measured for a subsample of approximately 10 trees per plot (h-trees) using Vertex III (Haglöf 2002) devices. To obtain the subsample, a basal area factor determined by the previous measurements of distances from plot center and dbhs, is used. In case of large changes or a new establishment of a plot, the basal area is determined using ACS before calipering trees. If 10 or fewer trees are within the plot radius, the heights of all trees are measured. The decision to measure 10 h-trees was made in 2005 (previously, three h-trees were measured), to provide better reference data for remote sensing applications. For the same reason, survey grade GNSS measurements of the plot coordinates are carried out by specialized field teams that do not measure other variables.

##### Prediction of volume and height

For each tree recorded on the 250 m^2^ plot, a height is first approximated using tree h-dbh functions (Vestjordet 1968; Fitje and Vestjordet 1978; Eid and Fitje 1993). Using dbh and the approximated height, volumes are predicted using allometric functions (Braastad 1966; Brantseg 1967; Vestjordet 1967; Bauger 1995). Volume functions for Norway spruce (Vestjordet 1967) are used for all spruce and fir species except for Sitka spruce (*Picea sitchensis* (Bong.) Carr.) where a separate function (Bauger 1995) is used. Volume functions for pine (Brantseg 1967) are used for all other coniferous trees and volume functions for birch (Braastad 1966) are used for all broadleaved species. For h-trees, tree volumes are additionally predicted using dbh and measured tree height. From the two volume predictions for h-trees, correction factors are calculated per sample plot and tree species group (spruce, pine, deciduous) as the ratio of the sum of tree volumes predicted using dbh and measured height and the sum of volumes predicted using dbh and approximated height. Each single tree volume is hereby inversely weighted with its angle-count selection probability such that trees with a smaller diameter and consequently a smaller selection probability have larger influence. If no trees with height measurements are available for a tree-species group on a sample plot, average correction factors given maturity class, site index class, tree species, and sampling region are used. Finally, the tree volume predicted for trees with no height measurements is multiplied by the correction factor. For h-trees, volumes are predicted using dbh and measured tree height. The heights of trees without height measurements are predicted by solving the volume function for height and using the predicted volume and dbh as input variables. Adjustments are made for breakages of stem parts with a diameter ≥ 10 cm. Above and below-ground biomass of conifers are predicted using single-tree allometric regression functions for spruce and pine developed in Sweden (Marklund 1988; Petersson and Ståhl 2006), while Norwegian birch functions (Smith et al. 2014, 2016) are used for the deciduous tree species.

##### Increment, mortality and harvest

The volume increment calculation is based on repeated diameter measurements. Assuming a growing season length of 100 days, the number of growing seasons is between 4.00 and 6.00 in a 5-year inventory cycle. The difference between the current and previous dbh is divided by the number of growing seasons to get a dbh 1 year before the current. For ingrown trees or in the case of establishing a new plot, the annual dbh differences are predicted from the average dbh of trees of the same tree species, diameter class, two maturity class groups, and site index. Volume 1 year before the current measurement is then predicted. Assuming the same diameter-height relationship as in the previous inventory cycle (see section before), a volume is predicted with the predicted dbh. Finally, the annual volume increment is the difference between the current volume and volume 1 year before.

The likely cause is recorded for broken or removed trees and the volumes of dead trees still present at the plot that were alive in the previous inventory cycle are predicted in the same way as for live trees. The volume of removed trees is predicted by adding 2.5 years of the previous increment to the previous volume prediction to account for the unobserved volume increment.

### Stand- and landscape-level variables on 0.1 ha plots

#### Land-use and land cover

Land-use and land cover (LULC) categories fully or partly covered by a sample plot are recorded. Because they do not belong to stand-level variables, we refer to them as landscape-level variables. For non-tree covered plots that are not visited in the field, they are identified using aerial images and other map data. LULC categories are mainly required for GHG reporting and a documentation of them can be found in the latest national inventory report (Norwegian Environment Agency 2019, Chapter 6). Except for houses (as part of settlements), all LULC categories must have an area of at least 0.1 ha to be identified as an independent class. If covered by trees, measurements on the 250 m^2^ plot are carried out on grasslands, wetlands, other land types, and some types of settlements that are accessible, but not in gardens, parks, and croplands.

#### The maturity class system

In productive forest, the maturity class of the stand is defined based on a combination of stand age, dominant tree species, and site index. This means, highly productive forests will move to an older maturity class at younger age than low-productive forests. Therefore, the age ranges of the maturity classes overlap if not separated by species and site index:

- Class 1: Forest under regeneration (age 0 yrs.)
- Class 2: Young forest (age 1–54 yrs.)
- Class 3: Young production forest (age 15–84 yrs.)
- Class 4: Older production forest (age 25–119 yrs.)
- Class 5: Mature forest (minimum age 40–120 yrs.)

A further division of the maturity classes 2–5 is made to describe whether the stand is sufficiently or poorly stocked, as defined by either the number of potential crop trees (class 2) or by basal area (classes 3–5). Furthermore, in stands with some scattered mature trees, basal area is used to determine whether a stand is to be considered a poorly stocked mature stand (class 5) or under regeneration (class 1).

#### Stand age

**Fig. 6:**
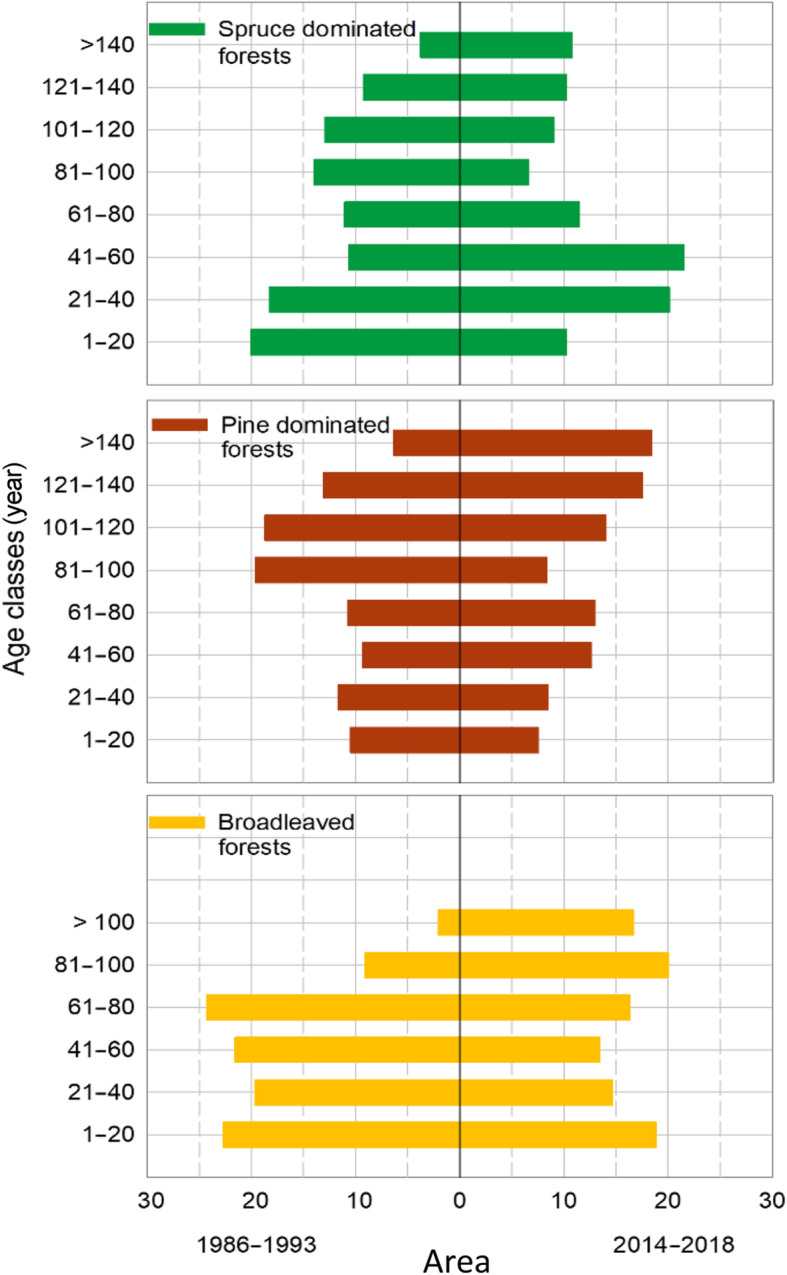
Distribution of productive forest area beneath the coniferous limit, by forest types and age classes for periods 1986–1993 and 2014–2018

Upon establishment of a permanent plot, stand age is determined from increment cores taken on one or more representative trees just outside the 250 m^2^-plot. The biological age, rather than chronological age, is recorded, by reducing for years of suppression below canopy after germination. Alternatively, the number of branch whorls is counted in young forest where this is possible. In forests that consist of either one or more than two layers, age is the basal-area weighted age of all trees. In two-layered forests, age is the basal-area weighted age of all trees in the overstory. Updates in subsequent inventory cycles are typically made by increasing the previously recorded stand age by another 5 years unless the stand has been harvested since the previous measurement. A new independent assessment must nonetheless be made in each inventory cycle. The new assessment may sometimes require a correction, e.g. if the stand has experienced substantial mortality or has been subject to harvests. The time series on stand age documents a clear trend towards an increased proportion of forest in the older age classes since the permanent plots were established (Fig. 6).

#### Site index

Since the 5th inventory, the H40-system (Tveite 1977; Tveite and Braastad 1981) is adopted, using dominant tree height at a base age of 40 years at breast height as the site index value. The measured site index is recorded as a class variable, using incremental steps of 3 m range where e.g. site index 14 corresponds to a dominant height between 12.5 and 15.5 m at 40 years breast height age. In cases of heterogenous soil conditions, i.e. where parts of the 0.1 ha plot area differ in productivity compared with the measured value, a subjective correction is made by the surveyor.

### Deadwood measurements along 18 m line transects

**Fig. 7:**
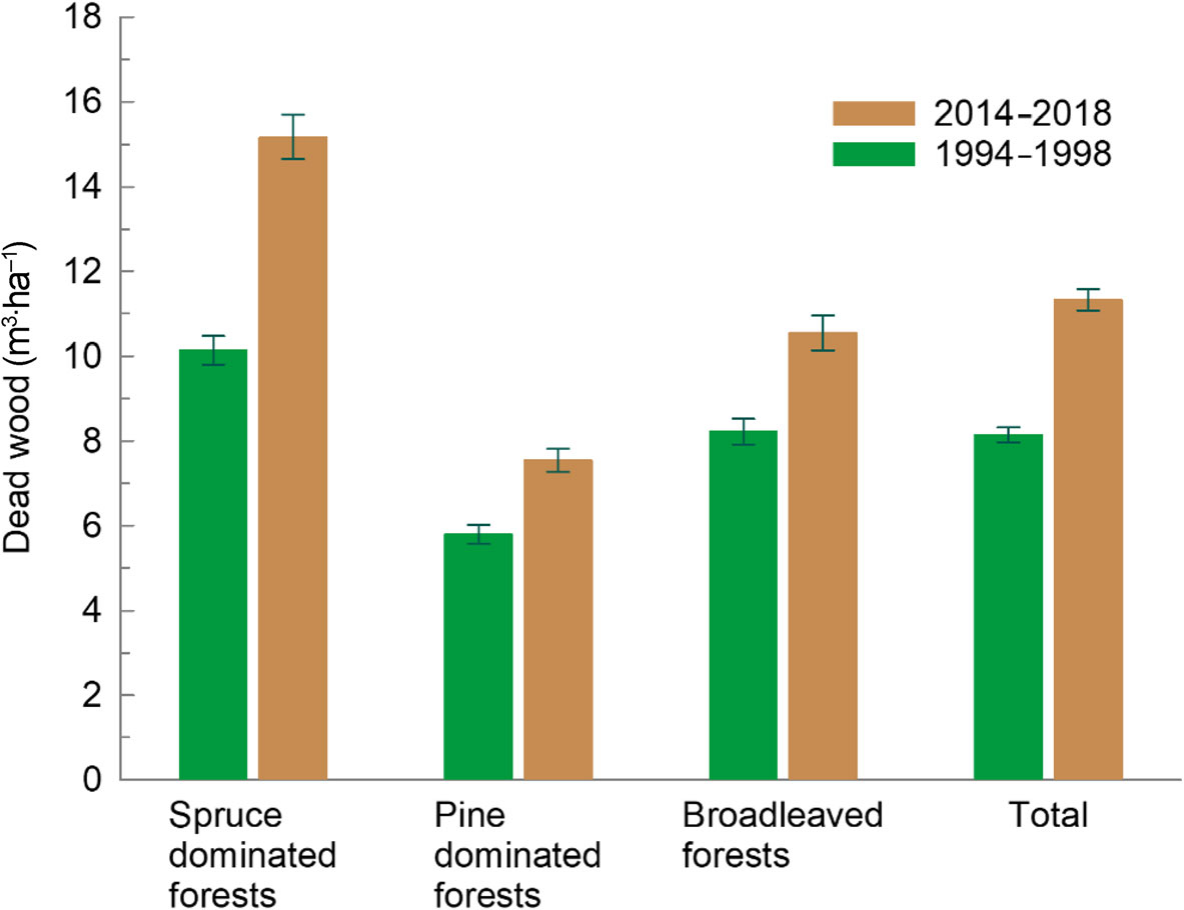
Amount of deadwood with diameter > 10 cm in productive forest beneath the coniferous limit, without Finnmark county, by forest types for periods 1994–1998 and 2014–2018. Whiskers represent 95% confidence intervals

During 1994–1998, downed deadwood was measured for the first time by assessing the full 250 m^2^ plot. In subsequent inventories the supply of new dead trees was recorded. Since 2010, the standing and downed deadwood volume are estimated using line intersect sampling (De Vries 1986, Chapter 13). In addition to decay status, species, length, and diameters at cross, root, and top of dead trees are measured. On more than 200 plots, both assessment methods were compared, but no significant difference in the resulting estimate was found. Since the first assessment, the amount of deadwood in productive forest has considerably increased (Fig. 7).

### Assessment of key habitats on 0.2 ha plots

In 2003, the Complementary Hot-spot Inventory methodology for mapping woodland key habitats (Gjerde et al. 2007), hereafter referred to as CHI habitats, was implemented in the NFI on 0.2 ha plots (Fig. 5). The results serve as a regional reference in the prioritization among candidate areas considered for retention, as required by certification schemes such as PEFC. To be defined as a CHI habitat, the area extent needs to be at least 0.2 ha and some fraction of the habitat needs to overlap with the 0.2 ha plot area. The area extent within the plot is mapped on paper in the field and is later digitalized into polygons at the office. Table 2 lists the main CHI habitats, their inclusion criteria and occurrence in productive forest. In addition to the listed habitats, selected topographical features (ravines, gorges, and steep rock walls) and objects (hollow deciduous trees) are included in the CHI habitat registrations (Viken 2018).

**Table 2:** Woodland key habitats recorded in the NFI since 2003, following the Complementary Hot-spot Inventory (CHI) methodology, with the main criteria for defining and mapping whole or parts of the 0.2 ha plot as a habitat polygon. The rightmost column gives the occurrence of the respective CHI habitats as of 2016 (inventory period 2014–2018)

CHI habitat	Main criteria for inclusion in polygon	Occurrence in productive forest (% of area)
Standing deadwood	• ≥ 40 standing dead trees·ha^−1^ with dbh ≥10 cm • Distance to other dead trees ≤15 m	2.7
Downed deadwood	• ≥ 40 downed dead trees·ha^−1^ with diameter at root end ≥10 cm • Distance to other downed dead trees ≤15 m	17.7
Trees with nutrient-rich bark	• Occurrence of maple (*Acer platanoídes* L.*)* or any tree species with bark lichens of the *lobarion* group • ≥ 20 trees·ha^−1^ of any of the above categories (60 in West and North Norway) • Distance to other trees with nutrient-rich bark ≤25 (15) m	0.3
Trees with pendant lichens	• ≥ 100 trees·ha^−1^ with at least 10 individuals/groups of pendant lichens of more than 10 cm length within 1 m^2^ of the vertical tree crown area • Occurrence of trees with lichens of the species *Evernia divaricata* (L.) Ach. or *Usnea longissima* Ach. • Only assessed for the lower 5 m of the tree	3.1
Deciduous boreal trees in late successional stage	• ≥ 40 trees·ha^−1^ with dbh ≥20 cm of any of the species *Populus tremula* L., *Sorbus aucuparia* L., *Alnus incana* L., *Salix caprea* L., *Prunus padus* L., or *Betula* spp. • Northern Norway: only *P. tremula* is considered	1.5
Old trees	• ≥ 30 standing old trees·ha^−1^ • Minimum age for spruce/pine = 150/200 yrs. • Deciduous trees are included dependent on dbh and species (minimum dbh is 30–40 cm for boreal species and 40–50 cm for noble hardwoods) • Distance to other old trees ≤20 m	2.5
Luxuriant ground vegetation	• Nutrient-rich forms of selected vegetation types sensu (Larsson 2005)	3.1

### Field work

Many variables recorded in the NFI require careful work, a lot of experience and training, and are partially or fully based on subjective estimates by the field staff. One example is stand age which is based on one or a few increment core measurements and subjective estimates of the basal area by stand layers. Other such examples are the estimation of bilberry and lingonberry cover, and the classification of land-cover types. The latter can be challenging for example in higher elevation areas where the landscape is often a rather fine-scaled matrix of productive and unproductive forest, and even with other land types blended in, such as other wooded land and areas without tree cover, and often with gradual transitions between the categories. Therefore, field crews are schooled for 1 week before every field season. At the time of writing, due to restrictions imposed by the Covid-19 situation, it is going to be the first year since the establishment of permanent plots without a field course. New fieldworkers are additionally schooled by an experienced supervisor for some weeks on the job, and each year the field workers are visited in the field by personnel from the office. The average experience of the fieldworkers is more than 10 years.

### Quality assurance

In addition to the annual field course and training of fieldworkers, several other measures are taken to assure a high data quality. Control inventories are done most years, which means that an experienced fieldworker is doing a “blind” registration of all variables on plots that have been measured by another person. While the main purpose is to identify whether certain variables may be understood and recorded differently by different persons, it also helps to identify systematic measurement errors due to human error or erroneous use of measurement devices. In turn, this may lead to a higher focus on selected variables during the subsequent field course, direct feedbacks to the fieldworkers, or to changes or additions to the NFI protocol. On average, about 2 % of the plots are subjected to such a control per year.

Data quality assurance is also aided by several logical tests in the data collection program in the field computer, allowing obvious measurement or punching errors to be corrected before leaving the plot. Upon the weekly transfer of data by email to the data repository, a control is also conducted by office personnel to identify likely errors, which includes a check of the new versus old data from the same plot. If likely errors or inconsistencies are identified at this stage, this may be communicated to the field crews, allowing a correction while the actual plot is still fresh in mind. After these initial routine checks, the data is stored temporarily in a separate database and later subjected to a more thorough and final quality control before being merged into the NFI database.

## Specialized inventories based on the NFI

### County inventories

Since the establishment of permanent plots in the sixth NFI (1986–1993, Section “The following NFIs until the 1990s”), additional temporary field plots are used to enable reliable estimates on county level approximately every 15 years. Until 2011, the temporary plots were concentric as in the sixth NFI in a cluster of up to 12 plots associated with the permanent plots. Since then, the measurement of dendrometric variables is also based on 250 m^2^ fixed area plots but the number of variables to measure is considerably reduced compared to the permanent plots. Due to the production of the Forest Resource Map SR16 (Section “Current developments and the foreseeable future”), the number of temporary plots was also considerably reduced.

### Inventory of protected areas

The environmental authorities need data on how the currently protected areas are distributed among different forest types to make sound priorities among candidate areas offered for protection by forest owners. Because more precise estimates were needed than could be provided using the existing grid of plots, additional permanent plots 1.5 km north and east of the existing permanent plots have been established since 2012 if they were located within a forest reserve (Astrup et al. 2011). A detailed report for the protected areas was published using these new data (Hylen et al. 2018). As the area of protected forest is currently increasing, new plots are continuously installed. Due to economic reasons, re-measurement of the plots that were established during 2012–2016 is currently limited to productive forest. To allow for a direct comparison of the forest conditions in reserves with other forest land, measurements follow the general NFI protocol.

## NFI use cases

### International reporting

NFI data are used for various international reporting requirements such as to FAO’s Forest Resource Assessment (MacDicken 2015) or to Forest Europe. However, no other international commitment has influenced the NFI design in the last 15 years more than the Climate Convention (UNFCCC) (UN 1992) and its Kyoto protocol. The NFI is the most important data source for the reporting in the LULUCF sector (Norwegian Environment Agency 2019, Chapter 6). The inclusion of areas above the coniferous forest line and Finnmark in the NFI are due to the UNFCCC reporting needs. Since the use of the NFI for reporting under the UNFCCC, other land use categories besides forest have been included or received more attention in the NFI and area estimates of land uses including settlements, agriculture, and wetlands, and changes among these, are reported using the NFI grid. Also new variables, such as the status of drainage on organic soils on all land use categories and the measurement of trees outside forest, have been implemented due to the reporting under the UNFCCC.

### Ecosystem services

Since the 1990s, a considerable number of new variables have been included in the NFI to accommodate the growing need of information about non-wood ecosystem services provided from forests, including biodiversity.

In the early 2000s, registration of important woody browsed species, such as rowan, aspen and willow, and browsing intensity from wild herbivores on trees with dbh < 5 cm was implemented, together with the assessment of bilberry cover. These data are annually reported to the Norwegian Monitoring Program for Cervids (Solberg et al. 2012) and have also recently been used to study whether the NFI estimates of woody browsing availability may be suitable for predicting browsing availability at local scales. Data from the bilberry cover registrations have been used to develop a model applicable to predicting the abundance of bilberry in Norwegian forest (Eldegard et al. 2019).

The many variables related to biodiversity and carbon accounting that have been implemented in the NFI since the 1990s have proven to be useful for a variety of purposes, in some cases even within a context that could not be foreseen at the time they were introduced. For example, data on deadwood and bilberry cover, and CHI habitats with old trees (Table 2), are currently used together with other indicators in the nature index for Norway (Nybø 2015), which is a framework for condensed reporting on the state of Norwegian nature. The nature index was launched in 2010 and is updated every 5 years. We believe that the NFI data related to biodiversity and other non-wood ecosystem services will see even more widespread use in the future, as the length of the time series increases.

### Research and the role of international cooperation

Besides the generation of forest statistics, the Norwegian NFI has also been an important basis for research. More than 60 scientific articles using NFI data have been published until today – the majority of them after 2000 (Tomter 2019, p. 170). Topics of those studies were among others the improvement of inventory methodology, biological models, and forest sector prognosis. Early research was rarely internationally published. For example, Alf Langsæter, one of the first NFI analysts, published several scientific reports in Norwegian (e.g. Langsaeter 1932, 1934). Among these was a work on uncertainty estimation in systematic strip sampling (Langsæter 1926) which contained early descriptions of estimators that are still studied today (Magnussen et al. 2020).

International cooperation was of great importance for the development of the Norwegian NFI. The first NFI report highlights the knowledge exchange with and influence of the Swedish NFI on the chosen sampling design (Landsskogtakseringen 1933, p.6). This close cooperation was maintained throughout the years, which is visible from similar developments in both NFIs (Fridman et al. 2014). The exchange with the other Nordic countries was, and still is, significant (Kangas et al. 2018). Cooperation through the European National Forest Inventory Network (ENFIN) and beyond has also been an important source of inspiration and quality assurance for the recent Norwegian NFIs (Tomppo et al. 2010).

## Current developments and the foreseeable future

### Forest growth and yield simulation and new variables

Providing forecasts on the development of the Norwegian forest has been an important task of the NFI, and prognoses, e.g. on sustainable yield, have been produced regularly during the past decades. Earlier prognosis tools (Eid and Hobbelstad 2000) were mainly used to forecast the development of timber resources given a set of assumptions on silvicultural strategies. Recent years have seen a growing need for a more flexible tool that may operate at the individual-tree level and accommodate also other ecosystem services such as carbon sequestration and the effects of climate change. Aided by advances in software and computational resources, this need has spurred a considerable effort in the development of a new open-source single-tree simulator during the last years. The developed simulator, named SiTree, is written in the R language for statistical computing (Antón-Fernández and Astrup 2019).

The simulator includes single-tree models for Norway and uses the soil model Yasso07 (Liski et al. 2005), such that also changes in soil carbon may be forecasted. Increment, mortality, and ingrowth of individual trees are forecasted either by a traditional empirical model-based approach or by imputation. In the latter, the future development of the focus tree is predicted by assigning the information of the nearest neighbor among the measured trees in the NFI database, using predictor variables from published empirical models to define the nearest neighbor tree. The simulator can flexibly accommodate a set of different silvicultural management options, different harvest pathways (Antón-Fernández and Astrup 2012), and changes in forest productivity due to changing climatic conditions (Antón-Fernández et al. 2016). Recently, the SiTree simulator has proven to be a valuable tool to analyze the effect of different climate mitigation measures in Norwegian forest (Bright et al. 2020, submitted) and in establishing a forest harvest reference level for Norway (Ministry of Climate and Environment 2019). Further development of the simulator will allow for even better tailored and more detailed scenario analyses in the years to come.

Classification of the NFI plots according to the Nature in Norway system (NiN) (Pedersen and Nybø 2015) was planned as of the field season 2020. Because the NiN system requires major additional field work and thus further qualification of the field staff, the implementation is currently postponed to 2021 due to the Covid-19 situation. Moreover, an initiative from the Ministry of Food and Agriculture and the Norwegian Environment Agency to elaborate on a soil sampling system may possibly lead to additions to the field protocol. While this would enable better parameterization of the Yasso soil carbon model and thus improving soil C estimates for Norway, it would require substantial resources for field sampling and subsequent laboratory analyses. Whether funding will be available for this task is still an open question.

### Utilization of remotely sensed data

Many users prefer maps over NFI statistics for administrative units because spatially-explicit information is (seemingly) easier to interpret. We notice that maps provide an intuitive access to data and the flexibility of aggregation for arbitrary regions. We therefore believe that the advantages of providing the required maps to users weighs stronger than local systematic errors that are inevitable in model-based maps. The NFI tries to satisfy these user needs with the Forest Resource Map SR16 (Astrup et al. 2019) which provides model-based raster maps of the most important variables in 16 m × 16 m resolution. Considerable research efforts towards the integration of remote sensing technologies in the NFI (Rahlf et al. 2014, 2015, 2017; Schumacher et al. 2020) have enabled its publication since 2015. As for numerous earlier developments of the NFI, SR16 and its satellite-based precursor (Gjertsen 2007) profited from the close knowledge exchange among the Nordic forest inventory community (Kangas et al. 2018) and the Nordic NFIs that provide similar systems to their users (Tomppo et al. 2008; Nord-Larsen and Schumacher 2012; Nilsson et al. 2017).

In SR16, metrics based on image matching and airborne laser scanning data are used to fit linking models for variables such as timber volume, biomass, and Lorey’s height, observed at the NFI sample plots. Dominant tree species are mapped using optical satellite data (Breidenbach et al. 2020) which improves the maps of other variables by enabling the stratification of models by tree species. The models are used to predict the variable of interest in the form of raster maps where remotely-sensed data are available. This means that the raster cells are the expected values of the models given the explanatory variables based on remotely sensed data. In addition to the expected values, maps of prediction intervals are provided as information on the uncertainty in the maps.

**Fig. 8:**
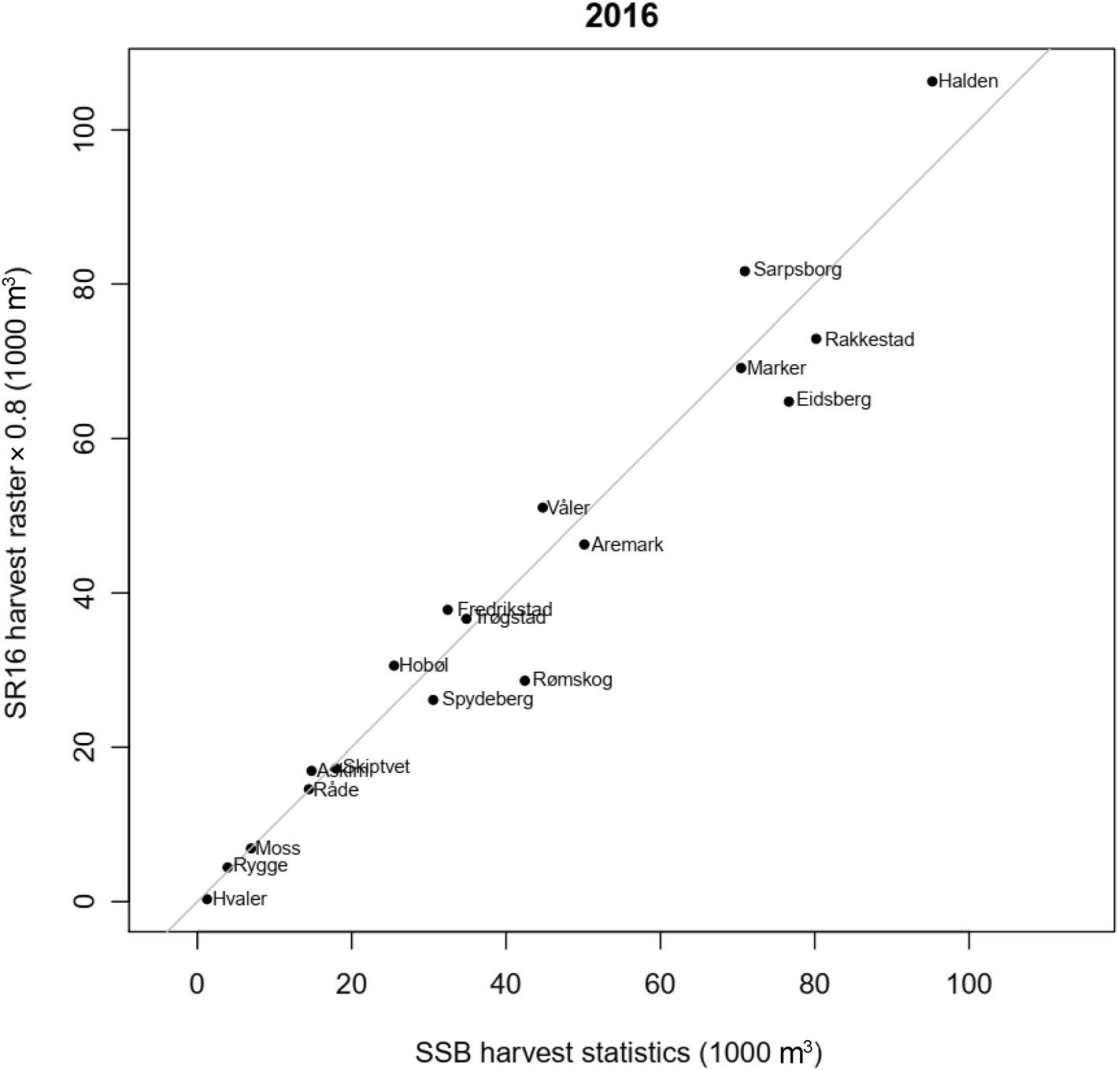
Synthetic estimates of harvested volume in 2016 assuming 80% of the standing timber volume is utilized per municipality for Østfold county based on the forest resource map SR16 vs. census statistics of wood harvested for industrial use data by Statistics Norway (SSB) for the same year

The SR16 maps are regularly updated for growth using linking models with updated parameter estimates using current NFI data. Changes due to larger removals such as harvests are updated by setting the predicted forest attributes in pixels with a Global Forest Watch change (Hansen et al. 2013; Rossi et al. 2019) to zero. This also provides harvested volume as a new map product. A comparison of synthetic estimates of SR16 harvest volume within several municipalities in south-eastern Norway with the official harvest statistics by Statistics Norway that is based on the mandatory reporting of harvested timber volume (Fig. 8) can also be seen as a form of validation of the SR16 volume map. In the synthetic estimates of harvest volume, it is assumed that 20% of the standing timber volume remains within the forest, which is based on analysis of NFI plots with harvests.

Owing to user requests, SR16 also provides synthetic estimates and their model-based uncertainties (Breidenbach et al. 2016, 2018) of the mapped (predicted) attributes for automatically generated segments that resemble forest stands. The segmentation and a parallel update of the official forest cover map (Ahlstrøm et al. 2019) is carried out by NIBIO’s Geomatics department, which also hosts SR16 as a freely available part of Norway’s natural resource infrastructure map server (Kilden 2020).

The NFI utilizes the remotely-sensed data prepared for SR16 in small area estimations on the municipality level (Breidenbach and Astrup 2012) which are disseminated to users by a web interface. The NFI web interface is available in English and Norwegian and gives access to standard NFI data as well (Breidenbach 2016–2020). Although alternative options are currently considered (Magnussen et al. 2020), conservative variance estimators assuming simple random sampling are utilized in the NFI web interface.

#### Future developments

Besides the further integration of remotely sensed data (Solberg et al. 2019; Puliti et al. 2020a, b), NFI researchers also work on improved tree-level assessments through terrestrial (Ducey and Astrup 2013; Astrup et al. 2014) or drone-based scanning techniques (Puliti et al. 2019, 2020a, b). We perceive the great potential of these relatively new measurement techniques in documenting the vegetation status, which enables new measurements at a later stage and provides additional information, for example on stem and crown shapes (e.g. Hyyppä et al. 2020). However, more research and development, especially with respect to all-weather capabilities and costs, will be needed to make them operational under the rough requirements of an NFI. Many of the variables assessed in the NFI will then still require careful assessment by field workers. A general tendency that can be read from the NFI development over the years is the need for more information. So far, the relatively simple design with fixed permanent plots for the primary variables has proven to be robust to the changing and increasing information requirements since its implementation in the 1990s. The fundamental societal changes required to form a fossil fuel-free bioeconomy without the loss of today’s welfare will require dramatic policy decisions. We are therefore confident that data provided by NFIs will be of high importance also in the future.

## Data Availability

Data are available upon reasonable request and from the cited repositories.

## References

[CR1] Aamlid D, Tørseth K, Venn K, Stuanes AO, Solberg S, Hylen G, Christophersen N, Framstad E (2000) Changes of forest health in Norwegian boreal forests during 15 years. Forest Ecol Manag 127(1–3):103–118

[CR2] Ahlstrøm A, Bjørkelo K, Fadnes KD (2019) AR5 Klassifikasjonssystem. NIBIO Bok. ISBN: 8217023255, NIBIO

[CR3] Anon (1914) Betänkande avgivet av kommissionen för försökstaxering av virkeskapital, tillväxt m.m. av skogarna i Värmlands län, Stockholm

[CR4] Antón-Fernández C, Astrup R (2012). Empirical harvest models and their use in regional business-as-usual scenarios of timber supply and carbon stock development. Scand J Forest Res.

[CR5] Antón-Fernández C, Astrup R (2019) SiTree: single tree simulator. CRAN https://CRAN.R-project.org/package=sitree. Accessed 4 May 2020

[CR6] Antón-Fernández C, Mola-Yudego B, Dalsgaard L, Astrup R (2016). Climate-sensitive site index models for Norway. Can J For Res.

[CR7] Astrup R, Ducey MJ, Granhus A, Ritter T, von Lüpke N (2014). Approaches for estimating stand-level volume using terrestrial laser scanning in a single-scan mode. Can J For Res.

[CR8] Astrup R, Rahlf J, Bjørkelo K, Debella-Gilo M, Gjertsen A-K, Breidenbach J (2019). Forest information at multiple scales: development, evaluation and application of the Norwegian forest resources map SR16. Scand J Forest Res.

[CR9] Astrup RA, Eriksen R, Antón-Fernández C, Granhus A (2011). Skogtilstanden i verneområder og vurderinger av mulighetene for intensivert overvåking gjennom Landsskogtakseringen.

[CR10] Barth A (1916). Norges skoger med stormskridt mot undergangen. Tidsskr Skogbr.

[CR11] Bauger E (1995). Volumfunksjoner og tabeller for furu, vanlig gran og sitkagran på Vestlandet.

[CR12] Bitterlich W (1948). Die Winkelzahlprobe. Allgm Forst u Holzwirts Ztg.

[CR13] Braastad H (1966). Volume tables for birch. Meddelelser fra det Norske Skogforsøksvesen.

[CR14] Brantseg A (1967). Volume functions and tables for Scots pine South Norway. Meddelelser fra det Norske Skogforsoksvesen.

[CR15] Breidenbach J (2016-2020) Landsskogtakseringen – Norway’s National Forest Inventory: make your own analyses. https://landsskog.nibio.no/. Accessed 1 Jun 2020

[CR16] Breidenbach J, Astrup R (2012). Small area estimation of forest attributes in the Norwegian National Forest Inventory. Eur J Forest Res.

[CR17] Breidenbach J, Magnussen S, Rahlf J, Astrup R (2018). Unit-level and area-level small area estimation under heteroscedasticity using digital aerial photogrammetry data. Remote Sens Environ.

[CR18] Breidenbach J, McRoberts RE, Astrup R (2016). Empirical coverage of model-based variance estimators for remote sensing assisted estimation of stand-level timber volume. Remote Sens Environ.

[CR19] Breidenbach J, Waser LT, Debella-Gilo M, Schumacher J, Rahlf J, Hauglin M, Puliti S, Astrup R (2020). National mapping and estimation of forest area by dominant tree species using Sentinel-2 data.

[CR20] Bright R, Allen M, Antón-Fernández C, Belbo H, Dalsgaard L, Eisner S, Granhus A, Kjønaas OJ, Søgaard G, Astrup R (2020) Evaluating the terrestrial carbon dioxide removal (tCDR) potential of large-scale aff−/reforestation and improved forest management in Norway. Glob Chang Biol, 10.1111/gcb.15228 10.1111/gcb.15228 32559355

[CR21] Bryn A, Daugstad K (2001). Summer farming in the subalpine birch forest. Man Biosphere Ser.

[CR22] Bryn A, Potthoff K (2018). Elevational treeline and forest line dynamics in Norwegian mountain areas–a review. Landsc Ecol.

[CR23] De Vries PG (1986) Sampling theory for forest inventory: a teach-yourself course. Springer-Verlag ISBN: 3642715818

[CR24] Ducey MJ, Astrup R (2013). Adjusting for nondetection in forest inventories derived from terrestrial laser scanning. Can J Remote Sens.

[CR25] Eid T, Fitje A (1993). Variations within stands for volume, basal area, number of trees, mean diameter and mean height. Meddelelser fra Skogforsk.

[CR26] Eid T, Hobbelstad K (2000). AVVIRK-2000: a large-scale forestry scenario model for long-term investment, income and harvest analyses. Scand J Forest Res.

[CR27] Eldegard K, Scholten J, Stokland JN, Granhus A, Lie M (2019). The influence of stand density on bilberry (*Vaccinium myrtillus* L.) cover depends on stand age, solar irradiation, and tree species composition. Forest Ecol Manag.

[CR28] Esmark HM (1965) Hogstklasser. In: Dyring T (ed) Landbrukets årbok. Skogbruk 1965. Et abonnements-oppslagsverk. Johan Grundt Tanum Forlag, Oslo

[CR29] Esser J (1994) Jordsmonn i bjørkeskog – en oversikt over Norge. NIJOS Rapp 4/94

[CR30] Esser J (1996) Kjemiske endringer i skogsjord på landsrepresentative overvåkingsflater etter 5-6 år. NIJOS Rapp 4/96

[CR31] Esser J, Nyborg Å (1992). Jordsmonn i barskog-en oversikt for Norge. NIJOS Rapp.

[CR32] Fitje A, Vestjordet E (1978). Bestandshøydekurver og nye hoydeklasser for gran. Meddelelser fra Norsk institutt for skogforskning.

[CR33] Fridman J, Holm S, Nilsson M, Nilsson P, Ringvall A, Ståhl G (2014). Adapting National Forest Inventories to changing requirements – the case of the Swedish National Forest Inventory at the turn of the 20th century. Silv Fenn.

[CR34] Gjerde I, Sætersdal M, Blom HH (2007). Complementary hotspot inventory – a method for identification of important areas for biodiversity at the forest stand level. Biol Conserv.

[CR35] Gjertsen AK (2007). Accuracy of forest mapping based on Landsat TM data and a kNN-based method. Remote Sens Environ.

[CR36] Haglöf A (2002) Users guide vertex III and transponder T3, Haglöf AB, Långsele

[CR37] Hansen MC, Potapov PV, Moore R, Hancher M, Turubanova SA, Tyukavina A, Thau D, Stehman S, Goetz SJ, Loveland TR (2013). High-resolution global maps of 21st-century forest cover change. Science.

[CR38] Helland A (1894). Skogenes fordeling inden elvenes nedslagsdistrikter. De norske flødningsvassdrag.

[CR39] Hylen G, Granhus A, Eriksen R (2018) Arealrepresentativ overvåking av skogvernområder gjennom Landsskogtakseringen. [Revidert] Rapport fra taksering utført i femårsperioden 2012-2016. NIBIO Rapport. 4

[CR40] Hyyppä E, Hyyppä J, Hakala T, Kukko A, Wulder MA, White JC, Pyörälä J, Yu X, Wang Y, Virtanen J-P (2020). Under-canopy UAV laser scanning for accurate forest field measurements. ISPRS J Photogramm Remote Sens.

[CR41] ICP Forest (2016). Manual on methods and criteria for harmonized sampling, assessment, monitoring and analysis of the effects of air pollution on forests. UNECE ICP Forests Programme Co-ordinating Centre.

[CR42] Ilvessalo Y (1927) Suomen metsät. Tulokset vuosina 1921-1924 suoritetusta valtakunnan metsien arvioimisesta. Commun Inst For Fenn 11

[CR43] Jonson T (1910). Taxaloriska undersökningar om skogsträdens form.

[CR44] Jonson T (1915). Massatabeller för träduppskattning utgifna.

[CR45] Kangas A, Astrup R, Breidenbach J, Fridman J, Gobakken T, Korhonen KT, Maltamo M, Nilsson M, Nord-Larsen T, Næsset E (2018). Remote sensing and forest inventories in Nordic countries–roadmap for the future. Scand J Forest Res.

[CR46] Kilden (2020) Skogressurskart SR16 - treslag [Forestresource map SR16 - tree species]. https://kilden.nibio.no/?lang=nb&topic=arealinformasjon&X=7117512.62&Y=315674.87&zoom=2&bgLayer=graatone_cache&catalogNodes=102,402,869&layers=skogressurs_treslag_beta&layers_opacity=0.75. Accessed 7 Apr 2020

[CR47] Kleinn C, Kändler G, Polley H, Riedel T, Schmitz F (2020) The National Forest Inventory in Germany: responding to forest related information needs. Allgemeine Forst und Jagdzeitschrift, AFJZ in press

[CR48] Kleinn C, Tomter S (1993). The Norwegian national forest inventory. Forstarchiv (Germany).

[CR49] Landsskogtakseringen (1920) Taksering av Norges skoger utført av Landsskogtakseringen - 1. Østfold fylke, Hamar

[CR50] Landsskogtakseringen (1933) Taksering av Norges skoger utført av Landsskogtakseringen, Oslo

[CR51] Landsskogtakseringen (1938) Taksering av Norges skoger utført av Landsskogtakseringen. Østfold fylke. Revisjonstaksering 1937, Oslo

[CR52] Landsskogtakseringen (1959) Taksering av Norges skoger utført av Landsskogtakseringen. Østfold og Akershus fylke. Revisjonstaksering 1957, Oslo

[CR53] Landsskogtakseringen (1968). The National Forest Survey of Norway - instructions for field work.

[CR54] Landsskogtakseringen (1970) Taksering av Norges skoger. Landsskogtakseringen 50 år. 1919–1969, Oslo

[CR55] Langsæter A (1926). Om beregning av middelfeilen ved regelmessige linjetaksering. Meddelelser fra det norske Skogforsøksvesen.

[CR56] Langsaeter A (1932). Nøiaktigheten ved linjetaksering av skog, I Bestemmelse av treantall og dimensjonsfordeling. Meddelelser fra det norske Skogforsøksvesen.

[CR57] Langsaeter A (1934). Nøiaktigheten ved linjetaksering av skog, II Bestemmelse av høide og årringbredde. Meddelelser fra det norske Skogforsøksvesen.

[CR58] Larsson JY (2005). Veiledning i bestemmelse av vegetasjonstyper i skog. Ny utgave 2005. NIJOS håndbok.

[CR59] Lindeberg JW (1924). Über die Berechnung des Mittelfehlers des Resultates einer Linientaxierung.

[CR60] Liski J, Palosuo T, Peltoniemi M, Sievänen R (2005). Carbon and decomposition model Yasso for forest soils. Ecol Model.

[CR61] MacDicken KG (2015). Global forest resources assessment 2015: what, why and how?. Forest Ecol Manag.

[CR62] Magnussen S, McRoberts RE, Breidenbach J, Nord-Larsen T, Ståhl G, Fehrmann L, Schnell S (2020). Comparison of estimators of variance for forest inventories with systematic sampling-results from artificial populations. Forest Ecosyst.

[CR63] Marklund L (1988). Biomassafunktioner för tall, gran och björk i Sverige (Biomass functions for pine, spruce and birch in Sweden), Department of Forest Survey.

[CR64] Ministry of Climate and Environment (2019). National forestry accounting plan for Norway for the first commitment period 2021–2025.

[CR65] Näslund M (1930). Om medelfelets beräkning vid linjetaxering. (On computing the standard error in line-survey). Svenska SkogsvFör. Tidskr.

[CR66] Nilsson M, Nordkvist K, Jonzén J, Lindgren N, Axensten P, Wallerman J, Egberth M, Larsson S, Nilsson L, Eriksson J (2017). A nationwide forest attribute map of Sweden predicted using airborne laser scanning data and field data from the National Forest Inventory. Remote Sens Environ.

[CR67] Nord-Larsen T, Schumacher J (2012). Estimation of forest resources from a country wide laser scanning survey and national forest inventory data. Remote Sens Environ.

[CR68] Nordli Ø, Wielgolaski F-E, Bakken AK, Hjeltnes SH, Måge F, Sivle A, Skre O (2008). Regional trends for bud burst and flowering of woody plants in Norway as related to climate change. Int J Biometeorol.

[CR69] Norwegian Environment Agency (2019) Greenhouse Gas Emissions 1990-2017. National inventory report. Norwegian Environment Agency, M-1271, Oslo

[CR70] Nybø S (2015) Beskrivelse av hovedøkosystemene og deres referansetilstand. Naturindeks for Norge. NINA, Trondheim

[CR71] Øyen K (2006). Kartlegginga av Noregs grøne gull: soga om Landsskogtakseringa, Jordregisterinstituttet og Norsk institutt for jord- og skogkartlegging frå 1919 til 2006.

[CR72] Pedersen B, Nybø S (2015). Naturindeks for Norge 2015 - Økologisk rammeverk, beregningsmetoder, datalagring og nettbasert formidling.

[CR73] Petersson H, Ståhl G (2006). Functions for below-ground biomass of *Pinus sylvestris*, *Picea abies*, *Betula pendula* and *Betula pubescens* in Sweden. Scand J Forest Res.

[CR74] Polley H (2020). Email subject: legal basis for NFI. European National Forest Inventory Network (ENFIN) email list.

[CR75] Puliti S, Breidenbach J, Astrup R (2020). Estimation of forest growing stock volume with UAV laser scanning data: can it be done without field data?. Remote Sens.

[CR76] Puliti S, Hauglin M, Breidenbach J, Montesano P, Neigh C, Rahlf J, Solberg S, Klingenberg T, Astrup R (2020). Modelling above-ground biomass stock over Norway using national forest inventory data with ArcticDEM and Sentinel-2 data. Remote Sens Environ.

[CR77] Puliti S, Solberg S, Granhus A (2019). Use of uav photogrammetric data for estimation of biophysical properties in forest stands under regeneration. Remote Sens.

[CR78] Rahlf J, Breidenbach J, Solberg S, Astrup R (2015). Forest parameter prediction using an image-based point cloud: a comparison of semi-ITC with ABA. Forests.

[CR79] Rahlf J, Breidenbach J, Solberg S, Næsset E, Astrup R (2014). Comparison of four types of 3D data for timber volume estimation. Remote Sens Environ.

[CR80] Rahlf J, Breidenbach J, Solberg S, Næsset E, Astrup R (2017). Digital aerial photogrammetry can efficiently support large-area forest inventories in Norway. Forestry.

[CR81] Rossi F, Breidenbach J, Puliti S, Astrup R, Talbot B (2019). Assessing harvested sites in a forested boreal mountain catchment through global forest watch. Remote Sens.

[CR82] Schumacher J, Hauglin M, Astrup R, Breidenbach J (2020). Mapping forest age using National Forest Inventory, airborne laser scanning, and Sentinel-2 data.

[CR83] Skogkommisjonen (1874). Indstilling med foreløbigt Udkast til Lov om Skovvæsenet fra Den ved Kongelig Resolution af 28de Marts nedsatte Kommisjon til Behandling af Skovlovgivningen.

[CR84] Smith A, Granhus A, Astrup R (2016). Functions for estimating belowground and whole tree biomass of birch in Norway. Scand J Forest Res.

[CR85] Smith A, Granhus A, Astrup R, Bollandsås OM, Petersson H (2014). Functions for estimating aboveground biomass of birch in Norway. Scand J Forest Res.

[CR86] Solberg EJ, Strand O, Veiberg V, Andersen R, Heim M, Rolandsen CM, Langvatn R, Holmstrøm F, Solem MI, Eriksen R (2012). Hjortevilt 1991-2011. Oppsummeringsrapport fra Overvåkingsprogrammet for Hjortevilt. NINA rapport.

[CR87] Solberg S, Andreassen K, Clarke N, Tørseth K, Tveito OE, Strand GH, Tomter S (2004). The possible influence of nitrogen and acid deposition on forest growth in Norway. Forest Ecol Manag.

[CR88] Solberg S, Kvaalen H, Puliti S (2019) Age-independent site index mapping with repeated single-tree airborne laser scanning. Scand J Forest Res 34:763-770

[CR89] Strand L (1994). Kilde til kunnskap. Landsskogtakseringen 75 år.

[CR90] Tewari V, Kleinn C (2015). Considerations on capacity building for national forest assessments in developing countries–with a case study of India. Int Forest Rev.

[CR91] Thorell K, Ostlin E (1931). The national forest survey of Sweden. J Forest.

[CR92] Tomppo E, Gschwantner T, Lawrence M, McRoberts RE (Eds.) (2010) National forest inventories. Pathways for common reporting. Springer.

[CR93] Tomppo E, Malimbwi R, Katila M, Mäkisara K, Henttonen HM, Chamuya N, Zahabu E, Otieno J (2014). A sampling design for a large area forest inventory: case Tanzania. Can J For Res.

[CR94] Tomppo E, Olsson H, Ståhl G, Nilsson M, Hagner O, Katila M (2008). Combining national forest inventory field plots and remote sensing data for forest databases. Remote Sens Environ.

[CR95] Tomter SM (2016) Norway. In: Vidal, C., Alberdi, I., Hernández, L., Redmond, J.J. (Eds.) National Forest Inventories - assessment of wood availability and use. Springer, pp 601–619

[CR96] Tomter SM (2019) Landsskogtakseringen 100 År 1919–2019. Norsk institutt for bioøkonomi-NIBIO, Ås

[CR97] Tomter SM, Hylen G, Nilsen J-E (2010) Norway. In: Tomppo E, Gschwantner T, Lawrencel M, McRoberts RE (eds) National Forest Inventories: pathways for common reporting. Springer, pp 411–424

[CR98] Tveite B (1977) Bonitetskurver for gran. Norsk Institutt for Skogforskning, vol 33

[CR99] Tveite B, Braastad H (1981) Bonitering for gran, furu og bjørk. Norsk Skogbruk, Norsk Institutt for Skogforskning, 27:17-22

[CR100] UN (1992) United Nations framework convention on climate change (UNFCCC), United Nations, New York 10.1111/resp.14705 38499332

[CR101] Vestjordet E (1967) Funksjoner og tabeller for kubering av stående gran (Functions and tables for volume of standing trees. Norway spruce). Meddelelser fra Det norske Skogforsøksvesen 22:539-574

[CR102] Vestjordet E (1968). Volum av nyttbart virke hos gran og furu basert på relativ høyde og diameter i brysthøyde eller ved 2,5 m fra stubbeavskjær. Meddelelser fra Det norske Skogforsøksvesen.

[CR103] Vidal C, Alberdi I, Hernández L, Redmond J (2016) National forest inventories - assessment of wood availability and use. Springer

[CR104] Viken KO (2018). Landsskogtakseringens feltinstruks 2018.

